# The Effect of Mandibular Angulation on Preoperative Assessment of Dental Implant Insertion at Premolar Region: CBCT Study

**DOI:** 10.1155/2022/7879239

**Published:** 2022-05-28

**Authors:** Sadaf Sadat Mahmoudinezhad, Azarnoosh AryanKia, Sanaz Sharifi Shooshtari, Kooshan Moradi

**Affiliations:** ^1^Department of Periodontics, School of Dentistry, Ahvaz Jundishapur University of Medical Sciences, Ahvaz, Iran; ^2^Department of Oral and Maxillofacial Radiology, School of Dentistry, Ahvaz Jundishapur University of Medical Sciences, Ahvaz, Iran

## Abstract

**Backgrounds:**

This study investigated the effect of mandibular angulation on the perioperative evaluation of the implant placement at the premolar region on panoramic reconstructed images using cone beam computed tomography (CBCT).

**Methods:**

A total of six dried anonymous human mandibles was included. Two implants were inserted in the left and right first premolar region. CBCT scans were obtained from each mandible at the standard position, +20° extension, and -20° flexion. The distance of the implant from the anterior loop of the inferior alveolar nerve and mental foramen was measured. Mean absolute error (MAE) of the distance of the implant from both anatomical landmarks was measured. The Wilcoxon matched-pair signed-rank test was used for the comparison of the measurements. All data were analyzed with the Stata program (version 15.1).

**Results:**

No statistically significant differences were found between the distance of the implant from the mental foramen and the anterior loop of the inferior alveolar nerve up to 20° extension and flexion at both sides of the mandible. (All *p* > 0.1) However, a variable range of MAE (SD) in the distance of the implant from different anatomical landmarks was found (0.9 ± 0.7 to 3.3 ± 2.1).

**Conclusions:**

We found no statistical difference in measurements of the distance of the implant from two anatomical landmarks at different head positions up to 20° extension and flexion. However, clinically, variable range in the distance of the implant from anatomical landmarks should be considered. Our findings could alert dentists of the possibility of error up to 20° extension and flexion on the perioperative evaluation of dental implant placement.

## 1. Introduction

Over the past decades, endosseous implants have become a common treatment for edentulous regions [1]. Inserting dental implants in the premolar region can be threatening for the mental foramen, mandibular incisive canal, and the anterior loop of the inferior alveolar nerve [2]. Therefore, some considerations must be taken to avoid violating these strategic anatomical landmarks during surgical procedures such as implant insertion [3].

Anatomically, the inferior alveolar nerve divides into two branches (anterior and posterior) at the anterior site of the trigeminal ganglion [4]. It enters the mandible through the mandibular foramen and travels to the mental foramen and may make a loop by running upward and backward before creating the mental foramen [5]. With regard to the mandibular canal, several investigations have noted that an extension of the mandibular canal called the mandibular incisive canal may locate mesial to the mental foramen, which can be in danger during implant insertion as well [6–8].

Inferior alveolar nerve injury during implant insertion may result in paresthesia, dysesthesia, and analgesia [9, 10]. Anterior region of the mental foramen could not be a 100% safe zone for implant insertion due to the presence of the aforementioned crucial anatomical landmarks [11]. Therefore, the anterior loop of the inferior alveolar nerve, the mental foramen, and the mandibular incisive canal should be carefully identified prior to implant surgery [7, 12].

In order to ascertain the precise location of the crucial anatomical landmarks such as anterior loop of the inferior alveolar nerve, the mental foramen, and the mandibular incisive canal, complete radiographic assessment should be obtained prior to surgery [7, 13]. However, it is noted in some previous studies that the identification of the mandibular incisive canal by conventional radiographies may be hard due to its less cortical bone and its manifestation as a labyrinth of intertrabecular spaces that contain neurovascular bundles [7, 11, 14].

Various imaging modalities have been introduced for presurgical implant assessment. Cone beam computed tomography (CBCT) has high clinical applications in the diagnosis and prediction of various dental diseases. It is also used in the clinical assessment of impacted teeth, diagnosis of temporomandibular disorders, pharyngeal airway assessment, and orthognathic surgery [15–18]. As an example, CBCT scans can provide detailed information about the place of the mandibular canal with lesser superimposition, magnification error, and image distortion, as compared to other imaging modalities such as panoramic radiographs [19]. Reliability on linear measurements in all spatial planes makes CBCT a reliable approach to attain more secure and reliable surgical plans, especially in implant surgeries [20].

Head orientation plays a paramount role in measurement accuracy during CBCT imaging; however, there are controversies about its impact on the measurement accuracy of CBCT [21–24]. CBCT manufacturers suggest following the reference lines while the occlusal plane of the mandible is parallel to the floor; however, patient's movements, patient disabilities, or inability to stay in a correct position causes discrepancies in image acquisition. Therefore, this may affect the identification of the precise location of the strategic landmarks [25, 26]. Misidentification at the premolar region in presurgical implant assessments leads to a violation of the crucial anatomical landmarks [27–31]. Although previous studies suggest a safety zone of 1 mm to 6 mm mesial to the mental foramen, there is no evidence if this range is reliable in case of patient's movements [3, 32, 33]. Therefore, accidental head deviation of the patient from the standard position before CBCT imaging may impact measurements for presurgical evaluation of the implant site insertion. CBCT equipment (e.g., NewTom VG CBCT instruments) uses software, which attempts to correct errors in head deviations during the reconstruction of study images from volumetric CBCT imaging.

This present study was aimed at investigating the effect of mandibular angulation on the assessment of the appropriate location for implant insertion at the premolar region on panoramic reconstructed images using NewTom CBCT imaging.

## 2. Methods

### 2.1. Radiographic Scan

A total of six (*n* = 6) dry anonymous human mandibles were selected randomly from the Head and Neck Radiology Department of Ahvaz Jundishapur University of Medical Sciences (AJUMS). The study was approved by the ethics committee of Ahvaz Jundishapur University of Medical Sciences (IR.AJUMS.REC.1398.526). The demographic information of dry human mandibles (age, gender, and ethnicity) was unknown to the researchers.

The skulls were selected based on having intact buccal and lingual cortical bones and alveolar ridges. Two implants with a height of 11.5 mm and a width of 4 mm were inserted in the left and right first premolar region. The mandibles were fixed on a sponge (20 cm∗20 cm∗20 cm) for reproducible angulations. There was no simulation for soft tissue to eliminate probable errors in measurements except the patients' motions.

**Figure 1:**
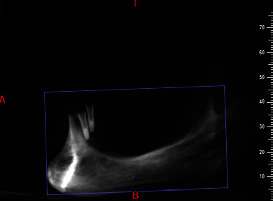
Standard position (gold standard).

**Figure 2:**
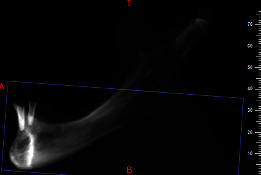
+20° extension: mandible was rotated 20° in the anterior side as compared to the standard position.

**Figure 3:**
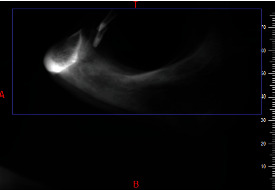
-20° flexion: mandible was rotated 20° in the posterior side as compared to the standard position.

The CBCT images were acquired from each mandible using NewTom VGi (NewTom VGi, QR Verona, Italy) at 110 KVP, 3.4 mA, 5.4 s, with the field of view of 8∗12 inch in three available image resolutions (voxel sizes = 0.150, 0.250, and 0.300 mm), and three positions as follows:

1. Standard position: mandibular base plane was parallel to the floor. This position was chosen as a gold standard (Figure 1)
2. +20° extension: mandible was rotated 20° in the anterior side as compared to the standard position. This position was created using a +20° wooden ramp, (Figure 2)
-20° flexion: mandible was rotated 20° in the posterior side as compared to the standard position. This position was created using a -20° wooden ramp (Figure 3)

### 2.2. CBCT Measurements

CBCT imaging was taken and evaluated twice with two-week intervals by two oral radiologists (SS and MR) with at least five years of experience. CBCT images were measured using NNT Viewer software (version 5.6, NewTom, Verona, Italy) and the same SONY VAIO VPCCW17FX Laptop Screen 14^″^.

**Figure 4:**
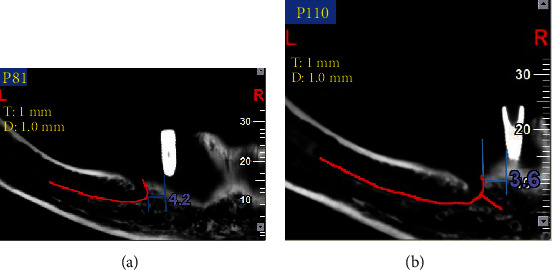
(a and b) The linear measurement between the anterior loop of the inferior alveolar nerve and the implant (red curve shows the inferior alveolar nerve path).

**Figure 5:**
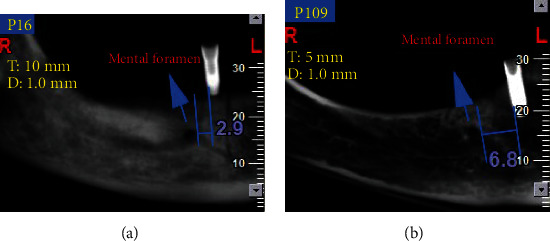
(a and b) The linear measurement between the mental foramen and the implant (blue arrow shows the mental foramen).

The panoramic images were reconstructed at 1 mm slice intervals with a 1 mm slice thickness. The distance between the implant and two anatomical landmarks at the premolar region (anterior loop and mental foramen) was measured. Two parallel lines were drawn across the implant surface and the anterior loop of the inferior alveolar nerve. The first line was drawn adjacent to the closest point of the implant to the anterior loop of the inferior alveolar nerve. The second line was drawn adjacent to the closest point of the anterior loop of the inferior alveolar nerve to the implant (Figure 4). The same method was used to measure the distance between the mental foramen and the implant (Figure 5). The distance between the two lines was measured in five positions as follows:

1. 0° (gold standard)
2. +20° extension, which was not corrected by NNT viewer autocorrection software
3. +20° extension, which was corrected by NNT viewer autocorrection software
-20° flexion, which was not corrected by NNT viewer autocorrection software
-20° flexion, which was corrected by NNT viewer autocorrection software

During CBCT imaging procedures, the observers could change the contrast, sharpness, resolution, slice thickness, and slice interval.

### 2.3. Statistical Analysis

Intraclass correlation coefficients for intraobserver and interobserver radiographic assessment reliability were measured. The outcome of interest was mean distance of the implant from the anterior loop and mental foramen in the right and left side of the mandible at gold standard position (radiographic measurements in the normal head position), -20° flexion (corrected and uncorrected), and +20° extension (corrected and uncorrected), respectively. The Wilcoxon matched-pair signed-rank test was used for the comparison of measurements at different angulations at each side of the mandible. Mean and standard deviations of absolute errors (MAE ± SD) for the distance of the implant from the mental foramen (right and left) and anterior loop of the inferior alveolar nerve (right and Left) at different angulations of the mandible were measured. The effect of different resolutions on the accuracy of linear measurements was evaluated in three available image resolutions. The significance level was set at *p* ≤ 0.05. Data were analyzed with the Stata program, version15.1. The methodology was reviewed by an independent statistician.

## 3. Results

The correlation between the measurements recorded by two examiners as testified from the ICC was significant (*p* < 0.001) and amounted to 0.9994 (95% confidence interval (CI) 0.9991-0.9996). The ICC between the measurements recorded at repeated sessions (reproducibility) was significant (*p* < 0.001) and amounted to 0.9992 (95% CI 0.9988-0.9994) for the first observer and 0.9995 (95% CI 0.9992-0.9996) for the second observer (Table 1).

Table 2 summarizes the mean distance of the implant from the right and left anterior loop of the inferior alveolar nerve; and right and left mental foramen at 0°(gold standard position), +20° (corrected and uncorrected), and -20° (corrected and uncorrected) in three different voxel sizes. The mean distance of implant from the right anterior loop, left anterior loop, right mental foramen, and left mental foramen at the gold standard position were 3.4 ± 2 mm, 4 ± 2.9 mm, 3.6 ± 1.9 mm, and 5.3 ± 2.6 mm, respectively, for the voxel size of 0.150 mm.

Table 3 shows the pairwise comparison between the distance of the implant from the right and left mental foramen and the right and left anterior loop of inferior alveolar nerves at different angulations of the mandible in similar resolutions. There were no statistically significant differences between the distance of the implant from the right anterior loop of the inferior alveolar nerve at different angulations (all *p* > 0.2). Likewise, there were no statistical differences between the distance of the implant from the left anterior loop of the inferior alveolar nerve at different angulations (all *p* > 0.2). Also, there were no statistically significant differences between the mean distance of implant from the mental foramen on the right side (all *p* > 0.1) and the left side of the mandible (all *p* > 0.3), respectively.

Table 4 shows mean and standard deviations of absolute errors for the distance of the implant from the mental foramen (right and left) and the anterior loop of the inferior alveolar nerve (right and Left) at different angulations of the mandible and in three different voxel sizes.

Supplementary Table 1 presents comparisons of the implant distance from the right and left anterior loop of the inferior alveolar nerve and the right and left mental foramen at similar mandibular angulation in different resolutions. No significant differences were found between the measurements in different resolutions at similar mandibular angulation (all *p* value > 0.1).

## 4. Discussion

In this study, we evaluated the linear measurement error by measuring the distance of the implant from the right and left anterior loop of the inferior alveolar nerve and right and left mental foramen at different angulations of the mandible, i.e., 0°, +20° extension (uncorrected and corrected by NNT view software), and -20° flexion (uncorrected and corrected by NNT viewer software). We showed no statistically significant difference between the measurements of the distance of the implant from the anterior loop of the inferior alveolar nerve (right and left) and mental foramen (right and left) at different angulations (All *p* value > 0.1). However, MAE of the distance of the implant from anatomical landmarks at different corrected and uncorrected angulations showed a variable range in right anterior loop (1.6 ± 2), left anterior loop (1.9 ± 3.3), right mental foramen (2.1 ± 2.6), and left mental foramen (0.9 ± 3.2). As far as we know, our study is the first study that assessed the effect of mandibular angulation on anatomical landmarks during implant insertion.

Several studies examined the accuracy of linear measurements by CBCT with different head positions using anatomical landmarks or opaque markers. For example, Sheikhi et al. [34] found that measurements of transverse distances among various anatomical landmarks (e.g., antegonion, jugale, zygomatic arch, width of nasal pyriform, zygomatico-frontal structure, and condylion) in central and in six other positions (10° and 20° tilts, 10° and 20° rotations, and 10° and 20° tips), in 3D reconstructed images, were underestimated compared to the actual values. However, these differences were not statistically significant. Furthermore, Kajan et al. [35] compared the height and width of 24 aluminum metal rods on three cast models. They found no statistically significant difference between the measurements up to 10° anteriorly and laterally tilted positions in cross-sectional images and autocorrected images by NNT viewer software from the NewTom VG CBCT device. Their results showed less than 0.5 mm difference compared with actual measurements. Shahidi and Feiz [36] also revealed that accuracy of transverse and vertical linear measurements between three radiopaque markers (marker 1: on the buccal area between first and second right mandibular molars at the level of alveolar crest, marker 2: at the level of marker 1, the same region at the lingual side, and marker 3: in line with marker 1 on the buccal side of the inferior border of the mandible) is maintained using Kodak 9000 CBCT in unintentional patient's movements up to 10° deviation from the standard position. Sheikhi et al. [34] compared physical buccolingual, mesiodistal, and height distances between opaque markers with those taken by Galileos CBCT in 5 different head positions (10-to-15-degree rotation, 10-to-15-degree tilt, 10-to-15-degree flexion, and 10-to-15-degree extension) and found 0.05 ± 0.45 mm difference between the standard position and deviated head position which was statically, but not clinically significant. El-Beialy et al. [21] similarly used Galileos CBCT for scanning one dry skull in 6 positions (central, posterior tilt, anterior tilt, lateral tilt, torsion, and complex). They used two methods to assess the accuracy of measurements. First, they compared 12 linear distances on the physical skull and the 3D virtual skull at the central and the other scanning positions. Then, registration of each of the five positions on the central position was done separately, and coordinates of 11 landmarks were identified in each position and compared with the central position. Concordance correlation and Pearson correlation coefficients values were almost 0.9999 in all the comparisons. Berco et al. [37] showed that skull orientation during CBCT scanning does not affect the accuracy or the reliability of 3-dimensional linear measurements using two positions (Frankfort horizontal parallel to the floor and the midsagittal plane perpendicular to the floor and Frankfort horizontal at approximately 45 to the floor and the midsagittal plane perpendicular to the floor). Tomasi et al. [38] measured 10 linear distances between different anatomical landmarks of a dry cadaver at two different positions (0° and tilted 45) and revealed no differences in linear measurements (overall absolute mean measurement error = 0.40 mm). Consistent with previous studies, Ludlow et al. [39] found that the accuracy of measurements was not significantly affected by alterations in skull position (ideal, shifted, and rotated) at the right or left sides on panoramic and three-dimensional images using NewTom 9000 CBCT and NewTom 3G software. Koch et al. [24] declared that the position of the mandible has a clinically insignificant effect on dimensional accuracy, up to 40° coronal and sagittal orientations. Consistent with the results of these studies, we did not find any statistically significant difference between the distance of implant from the right and left anterior loop of inferior alveolar nerve and right and left mental foramen at different corrected and uncorrected angulations.

Several studies reported differences in CBCT measurements at some positions before correction by CBCT software. Hassan et al. [22] showed no statistically significant difference between the ideal and the rotated (around the *Z* scanning axis by approximately 15–18 degrees) scan positions for the 3D images and the 2D tomographic slices using NewTom 3G CBCT, while statistically significant difference was observed between the ideal and rotated scan positions for the 2D projection images. Also, in this study, the radiographic measurements of the 3D images were closer to the physical measurements than the 2D slices and 2D projection images [22]. Kim et al. [23] used coordinates of the anatomical landmarks on the cadaver and concluded that improper patient's head position might result in measurement errors at 5° tilted and rotated positions. The difference in this study as compared to our study could be explained by using 3D image modality and RayScan Symphony® apparatus CBCT machine as compared to panoramic reconstructed images and NewTom CBCT used in our study. Also, we measured the linear distance between two definite points rather than the coordinates of the landmark. However, after autocorrection by OnDemand 3D™ software, there were no differences in distances by 5° deviation. Sabban et al. [26] measured the effect of different head positions on linear horizontal and vertical measurements at 7 different positions by 20° (extension, flexion, right lateral, left lateral, left flexion, and right flexion). They showed a significant difference in the vertical dimension, specifically at extension and flexion, which were more frequently seen in the posterior mandible. The observed differences were not reported after correction by in vivo dental CBCT reconstruction software. In contrast to this study, Adibi et al. [40] measured the shortest distance from the selected root apex to the superior border of the inferior alveolar nerve using NNT viewer software. They revealed no statistically significant difference in flexion and extension position. They found the difference at the laterally tilted head position by 15° on cross-sectional images.

The controversy among studies might be due to the different image modalities, intraoperator, and interoperator error. Nevertheless, there is no simulation which tracks closely to the real clinical situation, and human error in measurements is unavoidable in the clinical condition, since the operator measures the required distances in presurgical radiographic assessments.

In the present study, we did not measure physical distances on dry cadaver as several previous studies [21, 22, 34, 38]. We considered measurements at 0° angulation as a gold standard since CBCT measurements at 0° are reliably similar to physical measurements [41, 42]. Additionally, it seems that using anatomical landmarks (anterior loop of inferior alveolar nerve and mental foramen) as more realistic models of the anatomy could simulate the clinical situation, and they could provide us more reliable results as compared to the opaque markers used in some previous studies [34–36].

Although the differences in distance of implant from two anatomical landmarks (anterior loop of inferior alveolar nerve and mental foramen) were not statistically significant, we found a variable range of absolute mean error between implant and anatomical landmarks at flexion and extension (corrected and uncorrected) (Table 4). For example, the distance between implant from left anterior loop in -20° extension position (uncorrected) with the voxel size of 0.150 mm showed a MAE of 3.3 ± 2.1 mm which has a wide range of error in deviation from reference position. In addition, NNT viewer software decreased the absolute mean error in the distance of the implant from the anterior loop of the inferior alveolar nerve and mental foramen. The difference in measurements of the distance of implant from the anterior loop of the inferior alveolar nerve and mental foramen after correction by NNT viewer software was not statistically significant as compared to the reference position. Similar to uncorrected measurements, measurements after correction by NNT software showed a wide range of MAE. For example, the distance between implant from right mental foramen in 20° flexion position (corrected) showed a MAE of 2 ± 2.5 mm which has a wide range of error in deviation from the reference position. However, each millimeter is crucial during implant surgery; therefore, in clinical point of view, dentists should take into account the variable range of errors at different head positions for individual implant surgeries [26]. It is notable that following the CBCT scans in different voxel sizes (0.150 mm, 0.250 mm, and 0.300 mm), no statistical differences were found between the measurements in similar angulations. Our finding is consistent with previous studies implying that different available clinical resolutions do not affect the accuracy of the linear measurements [43–46].

The present study suffers from some limitations such as small number of dry human mandibles, difficulty in finding the anatomical landmarks on CBCT images, especially in the positions with -20° deviation, and disability in the measurement of physical distance between the implant and anterior loop of inferior alveolar nerve. Besides, different sections of CBCT imaging could be used to measure linear distances; for example, cross-sectional images can be used to measure bone length and width. In this research, implant and mental foramen or implant and inferior alveolar nerve are not vertically aligned (not below each other), so it may not be possible to find each of the landmarks simultaneously as the implant in a section of the cross-sectional photograph. Images with sagittal or reconstructed panoramic sections show a good horizontal relationship between the components. In this study, reconstructed panoramic images were used to measure the horizontal distance between the mental foramen and the anterior loop of the inferior alveolar nerve and the implant. Additionally, for the purpose of our study, we considered two easily identified landmarks (mental foramen and anterior loop of the inferior alveolar nerve) as two indices in the present study to evaluate the effect of different mandibular angulation on the linear distances between the implant and the anatomical landmarks. Therefore, we did not consider the mandibular incisive canal (MIC) as another index in the anterior mandibular region for measuring the distance between MIC and the implant. Since MIC becomes thinner while progressing mesially from the mental foramen to the mandibular anterior region, it may become challenging to be detected on CBCT, resulting in less accurate linear measurements (measurement bias) [47]. Also, there are variations in the visibility of MIC with different CBCT systems [47–49]. Finally, due to insufficient bone quantity and quality, detecting MIC in the edentulous mandible (used in the present study) is more difficult as compared to the dentate mandible [50]. Therefore, selecting MIC as an index for measuring the linear distances between the implant and MIC might not be a good choice to assess the effect of mandibular angulation on the presurgical assessment of implant insertion.

NewTom VGi CBCT showed statistically acceptable measurements of the distance of implant from mental foramen and anterior loop of the inferior alveolar nerve at different head positions up to 20° extension and flexion; however, clinically, variable range in distance of implant from anatomical landmarks should be considered during implant surgery. Our study could alert dentists the possibility of error in the accuracy of the presurgical assessment of appropriate implant site up to 20° head deviation in CBCT imaging for implant insertion during presurgical planning for implant insertion. This finding is crucial, especially for the elderly and other individuals who cannot keep their head in the correct position. The finding in this study should be investigated with more sample size in future studies.

## Figures and Tables

**Table 1:** Intraclass correlation coefficient for intraobserver and interobserver radiographic assessment reliability.

	Intraobserver				Interobserver	
	Observer 1	Significance (*p*)	Observer 2	Significance (*p*)		Significance (*p*)
Individual measures	0.9992 (0.9988-0.9994)	<0.001	0.9995 (0.9992-0.9996)	<0.001	0.9994 (0.9991-0.9996)	<0.001
Average measures	0.9996 (0.9994-0.9997)	<0.001	0.9997 (0.9996-0.9998)	<0.001	0.9997 (0.9996-0.9998)	<0.001

**Table 2:** Mean distance of the implant from the right and left anterior loop of inferior alveolar nerve and right and left mental foramen at 0° (gold standard position), +20° (corrected and uncorrected), and -20° (corrected and uncorrected) in three different voxel sizes.

Location/angulation	Voxel size	Mean ± SD (mm) (right AL)	Mean ± SD (mm) (left AL)	Mean ± SD (mm) (right MF)	Mean ± SD (mm) (left MF)
0°	(0.150 mm)	3.4 ± 2	4 ± 2.9	3.6 ± 1.9	5.3 ± 2.6
0°	(0.250 mm)	3.3 ± 2.1	4 ± 2.9	3.7 ± 1.9	5.3 ± 2.6
0°	(0.300 mm)	3.4 ± 2	4.1 ± 2.9	3.6 ± 1.9	5.4 ± 2.6
+20° corrected	(0.150 mm)	3.9 ± 2	4.5 ± 2.5	4.7 ± 2.2	5 ± 2.3
+20° corrected	(0.250 mm)	4 ± 2	4.5 ± 2.5	4.7 ± 2.3	5 ± 2.3
+20° corrected	(0.300 mm)	3.9 ± 2	4.5 ± 2.6	4.8 ± 2.4	5.1 ± 2.3
+20° uncorrected	(0.150 mm)	4.4 ± 2	4.5 ± 3.4	4.2 ± 2.2	5.4 ± 3.3
+20° uncorrected	(0.250 mm)	4.4 ± 2	4.4 ± 3.5	4.3 ± 2.3	5.4 ± 3.3
+20° uncorrected	(0.300 mm)	4.5 ± 2	4.5 ± 3.5	4.3 ± 2.3	5.5 ± 3.3
-20° corrected	(0.150 mm)	4.5 ± 2.3	4 ± 3	4.4 ± 2.1	4.6 ± 3.5
-20° corrected	(0.250 mm)	4.5 ± 2.2	3.9 ± 3	4.5 ± 2.2	4.7 ± 3.5
-20° corrected	(0.300 mm)	4.6 ± 2.3	3.9 ± 2.9	4.6 ± 2.2	4.8 ± 3.5
-20° uncorrected	(0.150 mm)	3.9 ± 3	2.7 ± 2	3.5 ± 2.7	3.8 ± 2.5
-20° uncorrected	(0.250 mm)	4 ± 3	2.7 ± 2	3.6 ± 2.8	3.8 ± 2.4
-20° uncorrected	(0.300 mm)	4 ± 3	2.7 ± 1.9	3.6 ± 2.7	3.8 ± 2.5

**Table 3:** Pairwise comparison between the distance of implant from mental foramen (right and left) and anterior loop of the inferior alveolar nerve (right and left) at different angulations of the mandible in similar resolutions (a: voxel size = 0.150 mm, b: voxel size = 0.250 mm, and c: voxel size = 0.300 mm).

Angulation/side	Implant from ^∗^AL (^∗^R), *p* value	Implant from ^∗^AL (^∗^L), *p* value	Implant from ^∗^MF (^∗^R), *p* value	Implant from ^∗^MF(^∗^L), *p* value
+20° corrected vs. 0°	0.7 (a)	0.6(a)	0.3(a)	0.6(a)
	0.6 (b)	0.5(b)	0.3(b)	0.6 (b)
	0.8 (c)	0.5 (c)	0.3(c)	0.6 (c)
+20° uncorrected vs. 0°	0.9(a)	0.8(a)	1.0(a)	0.8(a)
	0.9 (b)	0.9 (b)	0.9 (b)	0.8(b)
	0.9 (c)	0.9 (c)	0.9(c)	0.9(c)
+20° uncorrected vs. +20° corrected	0.5(a)	0.8(a)	0.4(a)	0.8(a)
	0.6 (b)	0.9 (b)	0.6 (b)	0.8(b)
	0.5 (c)	0.9(c)	0.6 (c)	0.9 (c)
-20° corrected vs. 0°	0.3(a)	0.7(a)	0.9(a)	0.8(a)
	0.5 (b)	0.8 (b)	0.9 (b)	0.8 (b)
	0.2(c)	0.8 (c)	0.8(c)	0.8 (c)
-20° corrected vs. +20° corrected	0.4(a)	0.8(a)	0.5(a)	0.7(a)
	0.5 (b)	0.8 (b)	0.6 (b)	0.5 (b)
	0.3 (c)	0.8(c)	0.7 (c)	0.5 (c)
-20° corrected vs. +20° uncorrected	0.8(a)	0.5(a)	0.6(a)	0.6(a)
	0.8 (b)	0.6 (b)	0.6(b)	0.6 (b)
	0.5 (c)	0.4 (c)	0.5 (c)	0.6 (c)
-20° uncorrected vs. 0°	0.9(a)	0.3(a)	0.9(a)	0.3(a)
	0.9 (b)	0.3 (b)	0.9 (b)	0.3(b)
	0.9 (c)	0.3(c)	0.9 (c)	0.3 (c)
-20° uncorrected vs. +20° corrected	0.9(a)	0.3(a)	0.2(a)	0.6(a)
	0.9(b)	0.3(b)	0.2 (b)	0.6 (b)
	0.9 (c)	0.3(c)	0.2 (c)	0.6 (c)
-20° uncorrected vs. +20° uncorrected	0.6(a)	0.2(a)	0.6(a)	0.5(a)
	0.6 (b)	0.2 (b)	0.6 (b)	0.5 (b)
	0.6 (c)	0.2 (c)	0.6 (c)	0.5(c)
-20° uncorrected vs. -20° corrected	0.3(a)	0.3(a)	0.1(a)	0.8(a)
	0.5 (b)	0.3 (b)	0.1 (b)	0.6 (b)
	0.3 (c)	0.3 (c)	0.1 (c)	0.6 (c)

∗AL: anterior loop; MF: mental foramen; R: right; L: left.

**Table 4:** Mean and standard deviations of absolute errors for the distance of the implant from the mental foramen (right and left) and the anterior loop of the inferior alveolar nerve (right and left) at different angulations of the mandible and in three different voxel sizes (a: voxel size = 0.150 mm, b: voxel size = 0.250 mm, and c: voxel size = 0.300 mm).

Location/angulation	^∗^MAE±^∗^SD (mm) (right ^∗^AL)	^∗^MAE±^∗^SD (mm) (left ^∗^AL)	^∗^MAE±^∗^SD (mm) (right ^∗^MF)	^∗^MAE±^∗^SD (mm) (left ^∗^MF)
+20° corrected	1.8 ± 2 (a)	2.1 ± 2.2 (a)	2.5 ± 2.1 (a)	0.9 ± 0.7 (a)
	2 ± 2 (b)	2.1 ± 2.2 (b)	2.5 ± 2.2 (b)	1 ± 0.7 (b)
	1.9 ± 1.9 (c)	2.2 ± 2.3 (c)	2.7 ± 2.2 (c)	1 ± 0.7 (c)
+20° uncorrected	2 ± 2.2 (a)	2 ± 1.7 (a)	2.2 ± 2 (a)	0.9 ± 0.8 (a)
	2 ± 2.3 (b)	1.9 ± 1.7 (b)	2.3 ± 2 (b)	1 ± 0.7 (b)
	2 ± 2.2 (c)	1.9 ± 1.8 (c)	2.4 ± 2 (c)	1 ± 0.8 (c)
-20° corrected	1.6 ± 2.1 (a)	1.9 ± 1.4 (a)	2.1 ± 2.1 (a)	2 ± 2.5 (a)
	1.6 ± 2.2 (b)	1.9 ± 1.3 (b)	2.1 ± 2.2 (b)	2.1 ± 2.5 (b)
	1.6 ± 2.2 (c)	2 ± 1.3 (c)	2.2 ± 2.3 (c)	2.2 ± 2.5 (c)
-20° uncorrected	1.9 ± 1.7 (a)	3.3 ± 2.1 (a)	2.6 ± 1.2 (a)	3.2 ± 2 (a)
	1.9 ± 1.7 (b)	3 ± 2 (b)	2.6 ± 1.3 (b)	3.2 ± 2 (b)
	1.8 ± 1.7 (c)	3 ± 1.9 (c)	2.5 ± 1.3 (c)	3.2 ± 2 (c)

∗MAE: mean absolute error; SD: standard deviation. ∗AL: anterior loop; MF: mental foramen.

## Data Availability

The data used to support the findings of this study are available from the corresponding author upon request (contact details: sadafmahmoudinezhad@gmail.com).

## Abbreviations

CBCT: Cone beam computed tomography
MAE: Mean absolute error.

## Additional Points

*Statement of clinical relevance*. Cone beam computed tomography provides reliable linear measurements in all spatial planes. However, head orientation plays a paramount role in measurement accuracy during CBCT imaging.

## Supplementary Materials

Supplementary Materials Supplementary Table 1: pairwise comparison of the implant distance from right and left anterior loop of the inferior alveolar nerve and right and left mental foramen at similar mandibular angulation in different resolutions (a: voxel size = 0.150 mm, b: voxel size = 0.250 mm, and c: voxel size = 0.300 mm).

## References

[1] Gómez-Román G., Lautner N. V., Goldammer C., McCoy M. (2015). Anterior loop of the mandibular canal—a source of possible complications. *Implant Dentistry*.

[2] Shaban B., Khajavi A., Khaki N., Mohiti Y., Mehri T., Kermani H. (2017). Assessment of the anterior loop of the inferior alveolar nerve via cone-beam computed tomography. *Journal of the Korean Association of Oral and Maxillofacial Surgeons*.

[3] Apostolakis D., Brown J. E. (2012). The anterior loop of the inferior alveolar nerve: prevalence, measurement of its length and a recommendation for interforaminal implant installation based on cone beam CT imaging. *Clinical Oral Implants Research*.

[4] Senger M., Stoffels H. J., Angelov D. N. (2014). Topography, syntopy and morphology of the human otic ganglion: a cadaver study. *Annals of Anatomy*.

[5] Alhassani A. A., AlGhamdi A. S. (2010). Inferior alveolar nerve injury in implant dentistry: diagnosis, causes, prevention, and management. *The Journal of Oral Implantology*.

[6] Jacobs R., Mraiwa N., vanSteenberghe D., Gijbels F., Quirynen M. (2002). Appearance, location, course, and morphology of the mandibular incisive canal: an assessment on spiral CT scan. *Dento Maxillo Facial Radiology*.

[7] Mraiwa N., Jacobs R., Moerman P., Lambrichts I., van Steenberghe D., Quirynen M. (2003). Presence and course of the incisive canal in the human mandibular interforaminal region: two-dimensional imaging versus anatomical observations. *Surgical and Radiologic Anatomy*.

[8] Barbosa D. A., Kurita L. M., Pimenta A. V. (2020). Mandibular incisive canal-related prevalence, morphometric parameters, and implant placement implications: a multicenter study of 847 CBCT scans. *Medicina Oral, Patología Oral y Cirugía Bucal*.

[9] Penarrocha M., Cervello M. A., Marti E., Bagan J. V. (2007). Trigeminal neuropathy. *Oral Diseases*.

[10] Renton T., Yilmaz Z. (2011). Profiling of patients presenting with posttraumatic neuropathy of the trigeminal nerve. *Journal of Orofacial Pain*.

[11] de Brito A. C., Nejaim Y., de Freitas D. Q., de Oliveira S. C. (2016). Panoramic radiographs underestimate extensions of the anterior loop and mandibular incisive canal. *Imaging Science in Dentistry*.

[12] Muinelo-Lorenzo J., Suarez-Quintanilla J. A., Fernandez-Alonso A., Varela-Mallou J., Suarez-Cunqueiro M. M. (2015). Anatomical characteristics and visibility of mental foramen and accessory mental foramen: panoramic radiography vs. cone beam CT. *Medicina Oral, Patología Oral y Cirugía Bucal*.

[13] Nagarajan A., Perumalsamy R., Thyagarajan R., Namasivayam A. (2014). Diagnostic imaging for dental implant therapy. *Journal of Clinical Imaging Science*.

[14] Polland K. E., Munro S., Reford G. (2001). The mandibular canal of the edentulous jaw. *Clinical Anatomy*.

[15] Walker L., Enciso R., Mah J. (2005). Three-dimensional localization of maxillary canines with cone-beam computed tomography. *American Journal of Orthodontics and Dentofacial Orthopedics*.

[16] Honey O. B., Scarfe W. C., Hilgers M. J. (2007). Accuracy of cone-beam computed tomography imaging of the temporomandibular joint: comparisons with panoramic radiology and linear tomography. *American Journal of Orthodontics and Dentofacial Orthopedics*.

[17] Aboudara C., Nielsen I., Huang J. C., Maki K., Miller A. J., Hatcher D. (2009). Comparison of airway space with conventional lateral headfilms and 3-dimensional reconstruction from cone-beam computed tomography. *American Journal of Orthodontics and Dentofacial Orthopedics*.

[18] Lo J., Xia J. J., Zwahlen R. A., Cheung L. K. (2010). Surgical navigation in correction of hemimandibular hyperplasia: a new treatment strategy. *Journal of Oral and Maxillofacial Surgery*.

[19] Angelopoulos C., Thomas S. L., Hechler S., Parissis N., Hlavacek M. (2008). Comparison between digital panoramic radiography and cone-beam computed tomography for the identification of the mandibular canal as part of presurgical dental implant assessment. *Journal of Oral and Maxillofacial Surgery*.

[20] Baumgaertel S., Palomo J. M., Palomo L., Hans M. G. (2009). Reliability and accuracy of cone-beam computed tomography dental measurements. *American Journal of Orthodontics and Dentofacial Orthopedics*.

[21] El-Beialy A. R., Fayed M. S., El-Bialy A. M., Mostafa Y. A. (2011). Accuracy and reliability of cone-beam computed tomography measurements: influence of head orientation. *American Journal of Orthodontics and Dentofacial Orthopedics*.

[22] Hassan B., van der Stelt P., Sanderink G. (2009). Accuracy of three-dimensional measurements obtained from cone beam computed tomography surface-rendered images for cephalometric analysis: influence of patient scanning position. *European Journal of Orthodontics*.

[23] Kim J. H., Jeong H. G., Hwang J. J., Lee J. H., Han S. S. (2016). The impact of reorienting cone-beam computed tomographic images in varied head positions on the coordinates of anatomical landmarks. *Imaging Science in Dentistry*.

[24] Koch G. K., Hamilton A., Wang K. (2019). Dimensional accuracy of cone beam CT with varying angulation of the jaw to the X-ray beam. *Dentomaxillofacial Radiology*.

[25] Spin-Neto R., Costa C., Salgado D. M., Zambrana N. R., Gotfredsen E., Wenzel A. (2018). Patient movement characteristics and the impact on CBCT image quality and interpretability. *Dentomaxillofacial Radiology*.

[26] Sabban H., Mahdian M., Dhingra A., Lurie A. G., Tadinada A. (2015). Evaluation of linear measurements of implant sites based on head orientation during acquisition: an ex vivo study using cone-beam computed tomography. *Imaging Science in Dentistry*.

[27] Mucci S. J., Dellon A. L. (1997). Restoration of lower-lip sensation: neurotization of the mental nerve with the supraclavicular nerve. *Journal of Reconstructive Microsurgery*.

[28] Deeb G. R., Dierks E., So Y. T. (2000). Sensory nerve conduction study of the mental nerve. *Muscle & Nerve: Official Journal of the American Association of Electrodiagnostic Medicine*.

[29] Willy P. J., Brennan P., Moore J. (2004). Temporary mental nerve paraesthesia secondary to orthodontic treatment — a case report and review. *British Dental Journal*.

[30] Morrison A., Chiarot M., Kirby S. (2002). Mental nerve function after inferior alveolar nerve transposition for placement of dental implants. *Journal of the Canadian Dental Association*.

[31] Zmener O. (2004). Mental nerve paresthesia associated with an adhesive resin restoration: a case report. *Journal of Endodontia*.

[32] Solar P., Ulm C., Frey G., Matejka M. (1994). A classification of the intraosseous paths of the mental nerve. *International Journal of Oral & Maxillofacial Implants*.

[33] Bavitz J. B., Harn S. D., Hansen C. A., Lang M. (1993). An anatomical study of mental neurovascular bundle-implant relationships. *International Journal of Oral & Maxillofacial Implants*.

[34] Sheikhi M., Ghorbanizadeh S., Abdinian M., Goroohi H., Badrian H. (2012). Accuracy of linear measurements of galileos cone beam computed tomography in normal and different head positions. *International Journal of Dentistry*.

[35] Kajan Z. D., Asli H. N., Taramsari M., Chai S. M. F., Hemmaty Y. B. (2015). Comparison of height and width measurements of mandibular bone in various head orientations using cone beam computed tomography: an experimental in vitro study. *Oral Radiology*.

[36] Shahidi S., Feiz A. (2013). Effect of minor amendments of patient’s position on the accuracy of linear measurements yielded from cone beam computed tomography. *Journal of Dentistry*.

[37] Berco M., Rigali P. H., Miner R. M., DeLuca S., Anderson N. K., Will L. A. (2009). Accuracy and reliability of linear cephalometric measurements from cone-beam computed tomography scans of a dry human skull. *American Journal of Orthodontics and Dentofacial Orthopedics*.

[38] Tomasi C., Bressan E., Corazza B., Mazzoleni S., Stellini E., Lith A. (2011). Reliability and reproducibility of linear mandible measurements with the use of a cone-beam computed tomography and two object inclinations. *Dento Maxillo Facial Radiology*.

[39] Ludlow J. B., Laster W. S., See M., Bailey L. T. J., Hershey H. G. (2007). Accuracy of measurements of mandibular anatomy in cone beam computed tomography images. *Oral Surgery, Oral Medicine, Oral Pathology, Oral Radiology, and Endodontology*.

[40] Adibi S., Shahidi S., Nikanjam S., Paknahad M., Ranjbar M. (2017). Influence of head position on the CBCT accuracy in assessment of the proximity of the root apices to the inferior alveolar canal. *Journal of Dentistry*.

[41] Stratemann S., Huang J., Maki K., Miller A., Hatcher D. (2008). Comparison of cone beam computed tomography imaging with physical measures. *Dentomaxillofacial Radiology*.

[42] Moshfeghi M., Tavakoli M. A., Hosseini E. T., Hosseini A. T., Hosseini I. T. (2012). Analysis of linear measurement accuracy obtained by cone beam computed tomography (CBCT-NewTom VG). *Dental Research Journal*.

[43] Costa A. L., Barbosa B. V., Perez-Gomes J. P., Calle A. J., Santamaria M. P., Lopes S. C. (2018). Influence of voxel size on the accuracy of linear measurements of the condyle in images of cone beam computed tomography: a pilot study. *Journal of Clinical and Experimental Dentistry*.

[44] Tolentino E., Yamashita F., De Albuquerque S. (2018). Reliability and accuracy of linear measurements in cone-beam computed tomography using different software programs and voxel sizes. *Journal of Conservative Dentistry*.

[45] Damstra J., Fourie Z., Huddleston Slater J. J., Ren Y. (2010). Accuracy of linear measurements from cone-beam computed tomography-derived surface models of different voxel sizes. *American Journal of Orthodontics and Dentofacial Orthopedics*.

[46] Kehrwald R., Castro H. S., Salmeron S., Matheus R. A., Santaella G. M., Queiroz P. M. (2021). Influence of voxel size on CBCT images for dental implants planning. *European Journal of Dentistry*.

[47] Ayesha R. T., Pachipulusu B., Govindaraju P. (2020). Assessment of prevalence and position of mandibular incisive canal: a cone beam computed tomography study. *Tzu-Chi Medical Journal*.

[48] Ramesh A., Rijesh K., Sharma A., Prakash R., Kumar A., Karthik (2015). The prevalence of mandibular incisive nerve canal and to evaluate its average location and dimension in Indian population. *Journal of Pharmacy & Bioallied Sciences*.

[49] Pires C. A., Bissada N. F., Becker J. J., Kanawati A., Landers M. A. (2012). Mandibular incisive canal: cone beam computed tomography. *Clinical Implant Dentistry and Related Research*.

[50] Sener E., Onem E., Akar G. C. (2018). Anatomical landmarks of mandibular interforaminal region related to dental implant placement with 3D CBCT: comparison between edentulous and dental mandibles. *Surgical and Radiologic Anatomy*.

