# Supplementary material for: The Effect of Mandibular Angulation on Preoperative Assessment of Dental Implant Insertion at Premolar Region: CBCT Study
Source: Biomed Res Int. 2022 May 28;2022:7879239. doi: 10.1155/2022/7879239 (PMC9167095; doi:10.1155/2022/7879239)
Supplement: Supplementary Materials — Supplementary Table 1: pairwise comparison of the implant distance from right and left anterior loop of the inferior alveolar nerve and right and left mental foramen at similar mandibular angulation in different resolutions (a: voxel size = 0.150 mm, b: voxel size = 0.250 mm, and c: voxel size = 0.300 mm). [file 7879239.f1.docx]

**Supplementary table1:** Pairwise comparison of the implant distance from right and left anterior loop of the inferior alveolar nerve and right and left mental foramen at similar mandibular angulation in different resolutions. ( a: voxel size= 0.150 mm , b: voxel size= 0.250 mm , c: voxel size= 0.300 mm)

| **Angulation/Resolution** | **Right AL^*^**  **P-value** | **Left AL**  **P-value** | **Right MF^*^**  **P-value** | **Left MF**  **P-value** |
| --- | --- | --- | --- | --- |
| 0° ( a vs. b) | 0.4 | 0.3 | 0.7 | 0.6 |
| 0° ( a vs. c) | 0.4 | 0.4 | 0.5 | 0.1 |
| 0° ( b vs. c) | 0.3 | 0.3 | 0.9 | 0.1 |
| +20°corrected  ( a vs. b) | 0.3 | 0.8 | 0.7 | 0.3 |
| +20°corrected  ( a vs. c) | 0.7 | 1.0 | 0.8 | 0.3 |
| +20°corrected  ( b vs. c) | 0.5 | 0.9 | 0.4 | 0.1 |
| +20°uncorrected  ( a vs. b) | 0.6 | 0.2 | 0.1 | 0.9 |
| +20°uncorrected  ( a vs. c) | 0.6 | 0.5 | 0.2 | 0.2 |
| +20°uncorrected  ( b vs. c) | 0.5 | 0.3 | 0.9 | 0.2 |
| -20°corrected  ( a vs. b) | 0.5 | 1.0 | 0.1 | 0.1 |
| -20°corrected  ( a vs. c) | 0.4 | 0.3 | 0.2 | 0.2 |
| -20°corrected  ( b vs. c) | 0.5 | 0.5 | 0.1 | 0.2 |
| -20°uncorrected  ( a vs. b) | 0.8 | 0.3 | 0.2 | 0.7 |
| -20°uncorrected  ( a vs. c) | 0.9 | 0.9 | 0.2 | 0.2 |
| -20°uncorrected  ( b vs. c) | 1.0 | 0.8 | 0.3 | 0.3 |

***AL: Anterior Loop; MF: Mental Foramen**
